# Examining gaze behavior in undergraduate students and educators during the evaluation of tooth preparation: an eye-tracking study

**DOI:** 10.1186/s12909-024-06019-4

**Published:** 2024-09-19

**Authors:** Frédéric Silvestri, Nabil Odisho, Abhishek Kumar, Anastasios Grigoriadis

**Affiliations:** 1 Department of Prosthodontics, School of Dental Medicine, ADES, CNRS, Aix-Marseille University, EFS Marseille, France; 2 Division of Oral Rehabilitation, Department of Dental Medicine, Karolinska Institutet, Huddinge, Sweden; 3 Division of Oral Rehabilitation, Department of Dental Medicine, Karolinska Institutet, Alfred Nobels Allé 8, Box 4064, 141 04 Huddinge, Sweden; 4 Academic Center for Geriatric Dentistry, Stockholm, Sweden

**Keywords:** Tooth preparation, Eye tracking technology, Undergraduate, Prosthodontics, Pilot study, Dental education

## Abstract

**Background:**

Gaze behavior can serve as an objective tool in undergraduate pre-clinical dental education, helping to identify key areas of interest and common pitfalls in the routine evaluation of tooth preparations. Therefore, this study aimed to investigate the gaze behavior of undergraduate dental students and dental educators while evaluating a single crown tooth preparation.

**Methods:**

Thirty-five participants volunteered to participate in the study and were divided into a novice group (dental students, *n* = 18) and an expert group (dental educators, *n* = 17). Each participant wore a binocular eye-tracking device, and the total duration of fixation was evaluated as a metric to study the gaze behavior. Sixty photographs of twenty different tooth preparations in three different views (buccal, lingual, and occlusal) were prepared and displayed during the experimental session. The participants were asked to rate the tooth preparations on a 100 mm visual analog rating scale and were also asked to determine whether each tooth preparation was ready to make an impression. Each view was divided into different areas of interest. Statistical analysis was performed with a three-way analysis of the variance model with repeated measures.

**Results:**

Based on the participants’ mean rates, the “best” and the “worst” tooth preparations were selected for analysis. The results showed a significantly longer time to decision in the novices compared to the experts (*P* = 0.003) and a significantly longer time to decision for both the groups in the best tooth preparation compared to the worst tooth preparation (*P* = 0.002). Statistical analysis also showed a significantly longer total duration of fixations in the margin compared to all other conditions for both the buccal (*P* < 0.012) and lingual (*P* < 0.001) views.

**Conclusions:**

The current study showed distinct differences in gaze behavior between the novices and the experts during the evaluation of single crown tooth preparation. Understanding differences in gaze behavior between undergraduate dental students and dental educators could help improve tooth preparation skills and provide constructive customized feedback.

## Background

The purpose of dental education programs is to impart undergraduate students with theoretical knowledge and to develop their motor and fine motor skills for effective management of dental procedures in different branches of dentistry. In most dental curriculums, students receive extensive pre-clinical and theoretical teaching to gain the ability to practice before taking on clinical cases. This allows students to master motor and fine motor skills necessary for effective management of dental procedures [1]. One of the main disciplines in dentistry is prosthodontics, defined by the Glossary of Prosthodontic Terms as “the dental specialty about the diagnosis, treatment planning, rehabilitation, and maintenance of the oral function, comfort, appearance, and health of patients with clinical conditions associated with missing or deficient teeth and/or maxillofacial tissues by using biocompatible substitutes” [2]. Although most dental students can easily acquire and validate theoretical knowledge before graduation, transforming this knowledge into practical motor skills remains complex for students and challenging for teachers to evaluate.

Preclinical courses provide an opportunity to assess undergraduates students abilities before they manage real clinical cases with patients [3]. However, dentists as well as health care practitioners need self-assessment skills or performance feedback to provide quality patient care. Self-assessment is described as an active process used by a student or a practitioner to objectively evaluate their knowledge, skills, and shortcomings to adapt and improve their skills [4, 5]. In the prosthodontic curriculum, theoretical knowledge allows undergraduates to identify areas of interest (e.g., finishing line, mesial-distal taper) when assessing a tooth preparation to make an objective self-assessment. Typically, a “feedback conversation” after a pre-clinical session could allow dental faculty to evaluate the student’s understanding by comparing their assessment with that of the educator [6]. Nevertheless, it has been shown that undergraduates tend to underrate or overrate their work, and therefore often do not improve significantly in their ability to self-evaluate [7]. Digital technologies such as intraoral scanners, software for evaluation of tooth preparation, virtual reality, etc., have offered newer tools to enhance the learning and motor skills of undergraduate students [8, 9]. Although undergraduate students self-assess their preclinical tooth preparation, it is difficult to identify common pitfalls in their evaluation method.

Recently, in other branches of medicine, eye-tracking technologies have been used to analyze and compare the gaze behavior of healthcare practitioners in different specialties [10–12]. Eye-tracking devices could also make it possible to have an objective assessment of areas of interest considered by the undergraduates for self-assessment. The eye tracking device is a sensor technology based on corneal reflection and stereo geometry. It allows a line-of-sight analysis by measuring different parameters of gaze behavior such as pupil diameter, number of fixations, duration of fixation, gaze path, and gaze location [12]. Moreover, this analysis could also provide information about unconscious behavior which cannot be obtained with a feedback conversation or another subjective tool such as a questionnaire [13]. In dentistry, few studies have utilized eye-tracking devices, with most focusing on analyzing visual perception [13–16] or interpreting radiographs [17, 18]. However, no study has yet reported the gaze behavior in the evaluation of undergraduate students’ evaluation of tooth-preparation. Therefore, the study aimed to investigate the gaze behavior in undergraduate dental students and dental educators while evaluating a single crown tooth preparation. It was hypothesized that there would be differences in gaze behavior, specifically reflected in shorter total duration of fixation, between undergraduate dental students and dental educators when evaluating a single crown tooth preparation.

## Methods

The participants of the study were groups of students and staff of the Department of Dental Medicine, Karolinska Institutet, Sweden. Written informed consents were obtained from all participants before participation in the study according to the Declaration of Helsinki. The project was approved by the Ethics Review Authority, Stockholm (Dnr 2023–04136-01).

### Study participants

Thirty-five participants volunteered to participate in the current observational study. Participants were divided into a novice group (*n* = 18, mean age = 22.9 ± 1.5; age range:22–28) consisting of undergraduate dental students in their seventh semester and an expert group consisting of dental educators (*n* = 17, mean age = 44.3 ± 13.0; age range: 30–74). Experts were dental educators with an average time since graduation of 19.0 ± 12.7 years and an average time in routine clinical practice of 16.7 ± 12.3 years. A power calculation was performed a priori using G*Power for an ANOVA with repeated measures and within-between interaction. For a medium effect size (f) of 0.3, an α error probability of 0.05, and a desired power of 0.90. the results indicated a required total sample size of 32 participants to achieve an actual power of approximately 0.91.

### Study setting

**Fig. 1:**
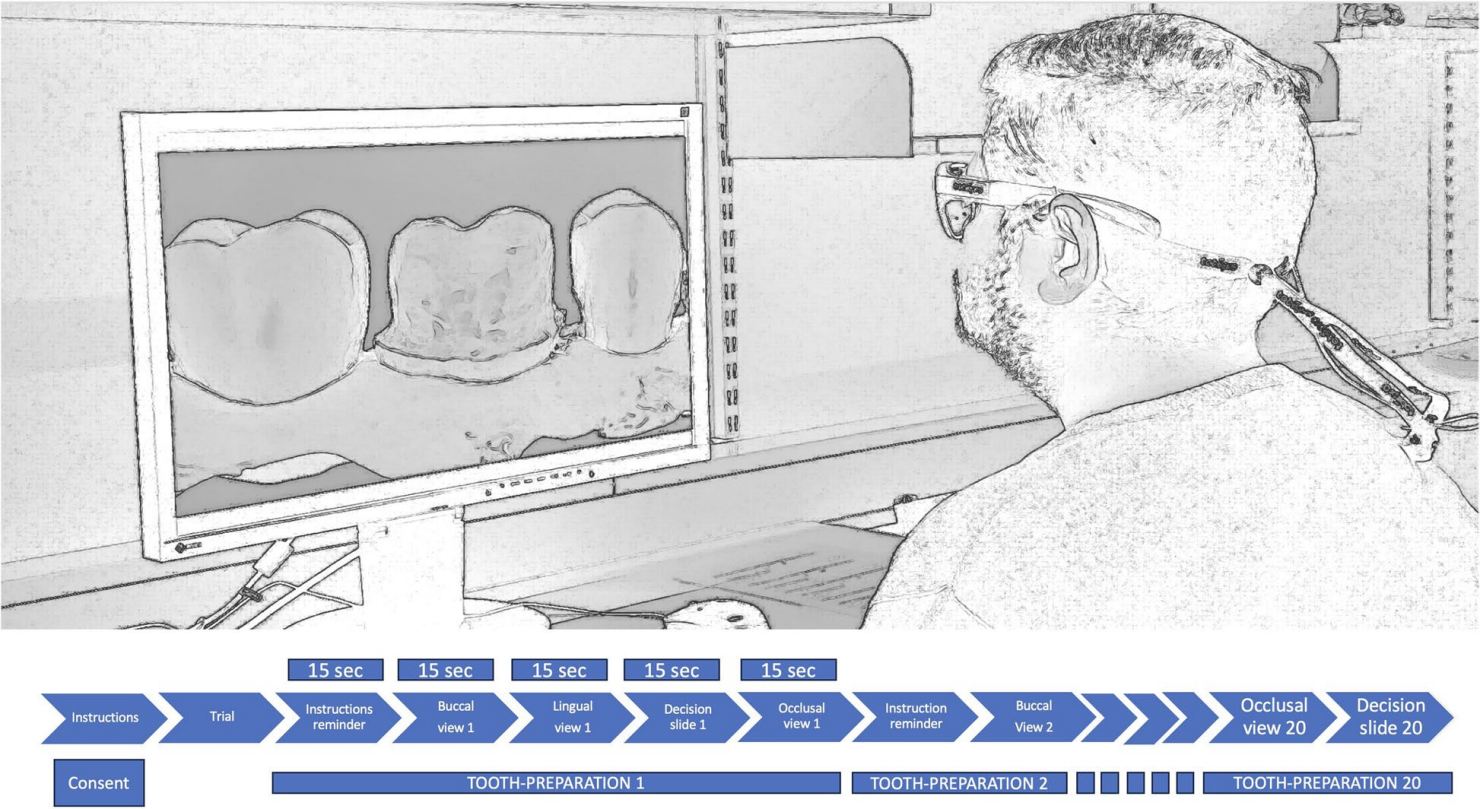
Showing experimental setup and timeline of the experimental session

The experiment has been designed following the Reporting Eye-tracking Studies In Dentistry (RESIDE) recommendations. [19] During the experiment, each participant was invited to participate in a signal experimental session of about 30 min. The participants (both groups) were asked to comfortably sit on an office chair in a well-lit quiet room illuminated with regular artificial light (3000 K). A screen was placed on a desk in front of a white wall (FlexScan® EV2416W, 24.1 inches,1920 × 1080 pixels, 50–60 Hz; Eizo Corporation, Japan). The height of the chair on which the participants were seated was adjustable and was about 0.75 to 1.0 m from the screen. The participants were asked to adjust the chair so that they could look horizontally at the screen. Each participant wore a binocular eye-tracking device (Tobii Pro Glasses 3®, Danderyd, Stockholm, Sweden). The participants were assisted by the examiner to carefully secure the wearable eye tracker like a spectacle. This eye-tracking system uses a one-point calibration procedure and has a gaze position accuracy of 0.6°. Participants were also asked to wear earplugs during the experiment to ensure maximum silence. Video recordings were carried out using dedicated software (Glasses 3 controller®, Danderyd, Stockholm, Sweden: Tobii AB) (Fig. 1). During the experiment, the examiner remained inside the room although away from the direct vision of the participants and observed the smooth conduct of the entire experimental process as discretely as possible.

### Selection and display of images

The examiner (FS) prepared twenty samples of acrylic, right first maxillary molars (Frasaco®, Franz Sachs GMBH & Co, Germany) for monolithic zirconia crowns. After satisfactory preparation, these samples were scanned using an intra-oral scanner (Cerec® Omnicam, Dentsply Sirona, Charlotte, United States). Subsequently, a software-supported evaluation of tooth preparations was conducted using Prepcheck® (Dentsply Sirona, Charlotte, United States), and all reports were obtained. From the scan files, three high-resolution images (1920 × 1080 pixels) were selected for each of the twenty-tooth preparation, showcasing a buccal, lingual, and occlusal views. This resulted in a total of twenty sets of images, each set containing three views of a single tooth preparation, amounting to a total of sixty images. These sixty images, representing twenty different tooth preparations in three views each, were displayed during the experimental session.

### Experimental protocol

Participants received verbal and written instructions explaining how the experimental session would be conducted. The participants were also briefly informed about the main objectives of the study. The participants were then asked to rate all twenty sets of tooth preparations (60 pictures in total) on a 100 mm visual analog rating scale (VAS) without landmarks from “very bad” to “very good”. Additionally, the participants were asked to respond to the question “Is this tooth preparation ready for making an impression for a monolithic zirconia crown?” by choosing the answers as “yes” or “no.”

**Fig. 2:**
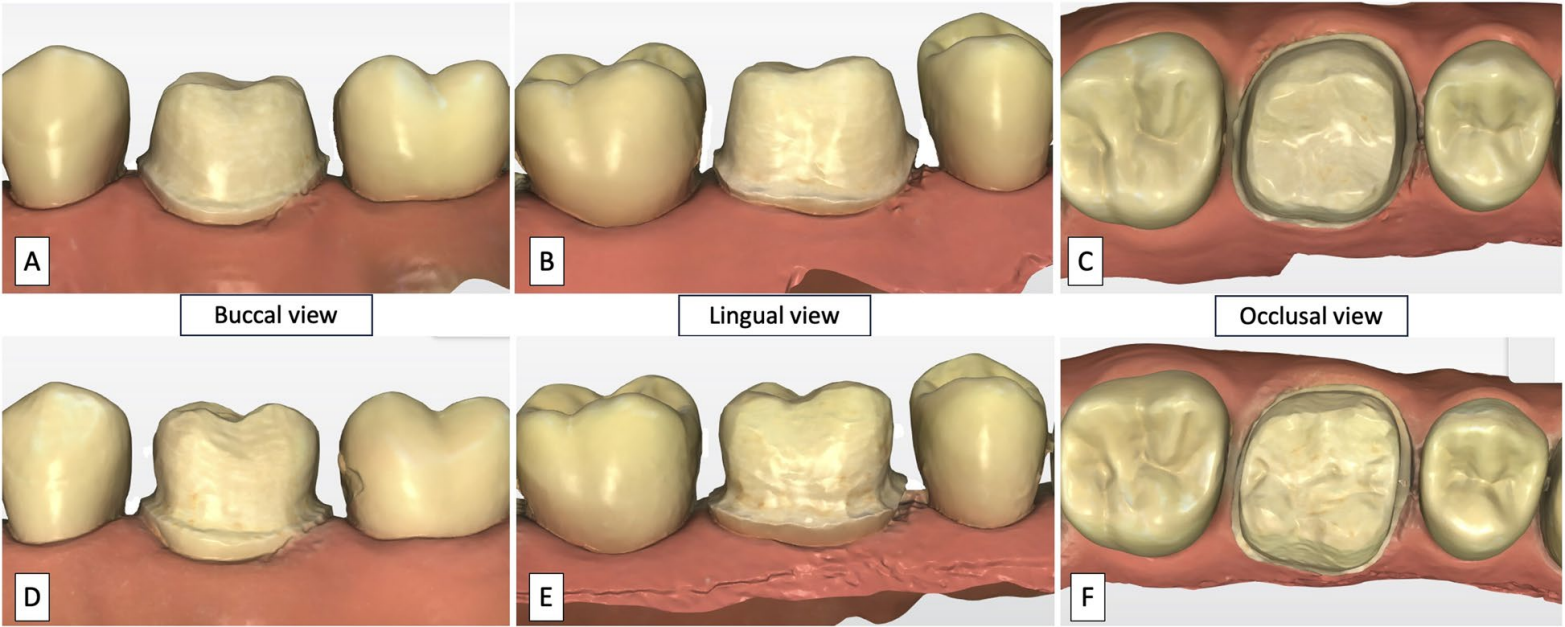
Examples of tooth preparation images from the buccal, lingual, and occlusal views, showcasing the best (**A**, **B**, **C**) and worst (**D**, **E**, **F**) preparations

All participant first performed a "test trial" to ensure they understood the instructions correctly. Then, the twenty sets of different tooth preparations, each with buccal, lingual, and occlusal views, were presented to the participants one by one on the computer screen (Fig. 2). Although all twenty sets of images were randomly arranged, the order in which they were assessed was the same for all participants. For each set, the buccal image was displayed first, followed by the lingual and then the occlusal view. Each image (buccal, lingual, or occlusal view) was displayed for 15 s. At the end of the three views (one set), participants had 15 s to rate the preparation on a 100 mm analog rating scale. They also responded to whether they thought the tooth preparation was ready for making an impression for a monolithic zirconia crown on a subject-based feedback form. Please note that the participants could move on to the next slide/picture if they thought they had made their decision with the 3 views of each tooth preparation before their allocated time of fifteen seconds. The participants were also given a break of one minute after five sets of the tooth preparations were displayed.

### Data analysis

**Fig. 3:**
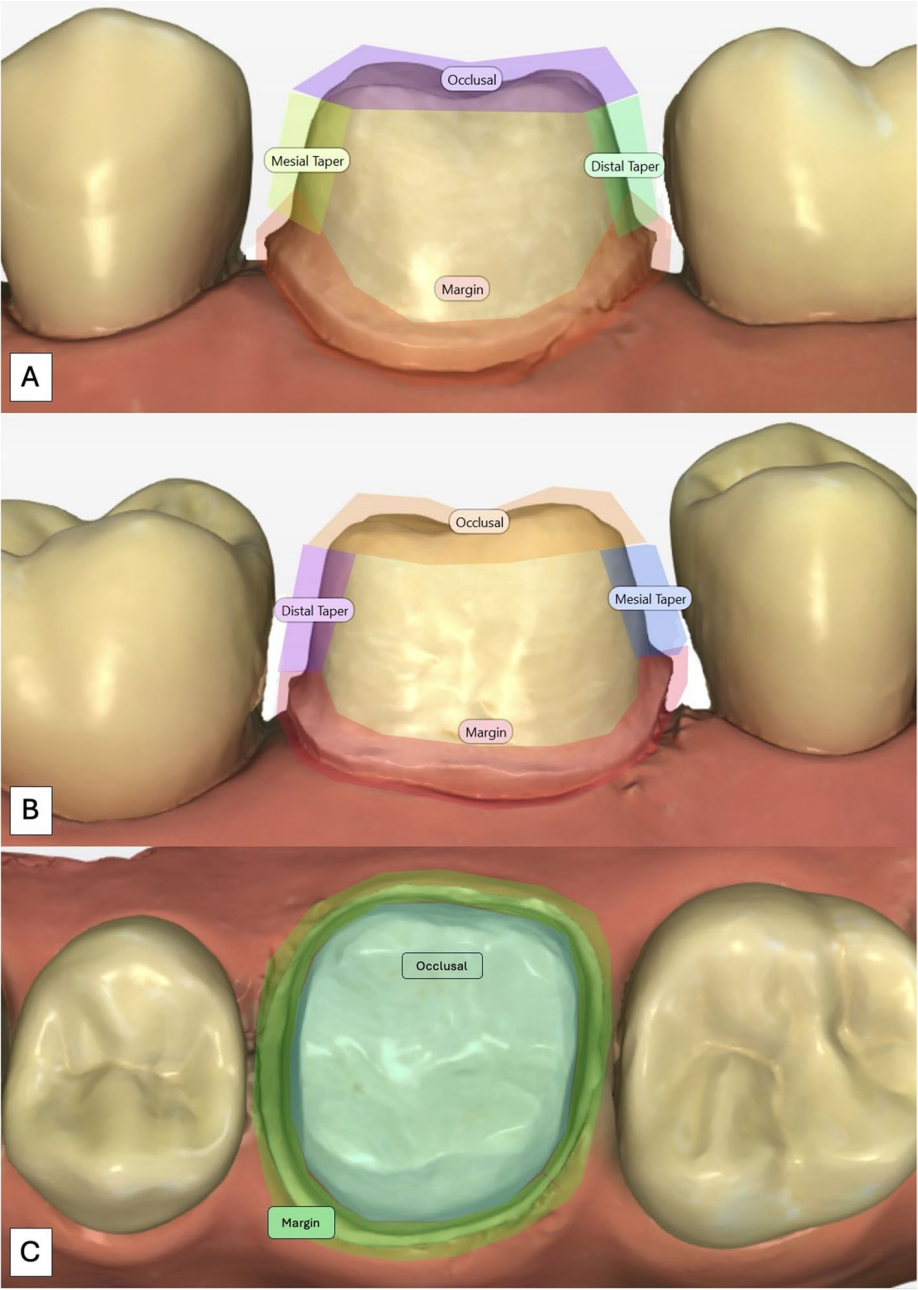
Showing different areas of interest drawn on buccal (**A**), lingual (**B**), and occlusal (**C**) views

For each participant, answers were recorded (yes = 1, no = 0) and scores of all tooth-preparations were collected by measuring the mark on the line (0 to 100). All the collected video files were then analyzed using a dedicated software (Tobii Pro Lab®, v 1.217, [Computer software]; Danderyd, Stockholm, Sweden: Tobii AB). Both buccal and lingual views were divided into four areas of interest (AOI), which included the margin, mesial taper, distal taper, and occlusal shape. Occlusal views were divided into two AOI, which included the margin and the occlusal area only. (Fig. 3) Each AOI was outlined in the software and the fixation threshold was set at 200 ms. First, the data was automatically mapped using a dedicated software (Tobii Pro Lab®). However, each gaze fixation was then manually checked by the examiner. For each AOI, the values of the total duration of fixation were analyzed as a metric of gaze behavior.

### Statistical analysis

The data was analyzed with SPSS (Statistical Package for the Social Sciences), version 27, IBM, inc. The data was checked for the assumptions of normal distribution with the Shapiro-Wilks test, histograms, and QQ plots. The scores of the acceptable and unacceptable tooth preparations were compared between the groups with the Wilcoxon Mann–Whitney U test. Further the dichotomous (yes/no) response to the question “Is this tooth preparation ready for making an impression for a monolithic zirconia crown?” were compared between the two groups with the Chi-Square test.

The total duration of fixations for the different views was evaluated with a three-way analysis of variance (ANOVA) model with repeated measures to analyze the different outcome parameters. Since the distribution of the variables was skewed the variables were log-transformed before subjecting to the repeated measures ANOVA. To avoid the loss of zero values, a small constant was added to all the variables before their logarithmic transformation [10]. The factors in ANOVA were groups (two levels: novices and experts), photos (best and worst preparation), and conditions (margin, mesial taper, distal taper, occlusal). Similarly, the duration of assessment for the different views was evaluated with a three-way analysis of variance (ANOVA) model with repeated measures. The factors in ANOVA were groups (two levels: novices and experts), photos (best and worst preparation), and views (buccal, lingual, and occlusal). Post hoc analysis of the significant main effects was done with the Unequal N HSD test. A *P* value of < 0.05 was considered statistically significant.

## Results

All participants completed the entire experimental session without any difficulty. The mean scores from all the participants for all the twenty tooth preparations were averaged and the “best” and the “worst” tooth preparations were selected for analysis. The novices rated 74.7 ± 17.8 and the experts’ 69.9 ± 20.5 for the best tooth preparation on the 100 mm visual analog scale. Similarly, the novices rated 22.1 ± 18.5 and experts 11.9 ± 15.0 for the worst tooth preparation on a 100 mm visual analog scale. However, there was no significant difference in the visual analog scale ratings for either the best (*P* = 0.478) or worst (*P* = 0.074) tooth preparation between the novices and experts.

### Subject-based reports

Subject-based reports further showed that about 77.8% of the participants in the novices and 64.7% of participants in the experts judged the best tooth preparation as the one “ready for making an impression for a monolithic zirconia crown.” While none of the participants in the experts agreed that the worst tooth preparation was ready for impression about 11.1% of the participants in the novices thought that it was still acceptable that the otherwise worst tooth preparation was ready for making an impression. However, there was also no significant correlation between the groups and their decision while judging either the best (*P* = 0.392) or worst (*P* = 0.157) tooth preparation.

### Buccal view

**Fig. 4:**
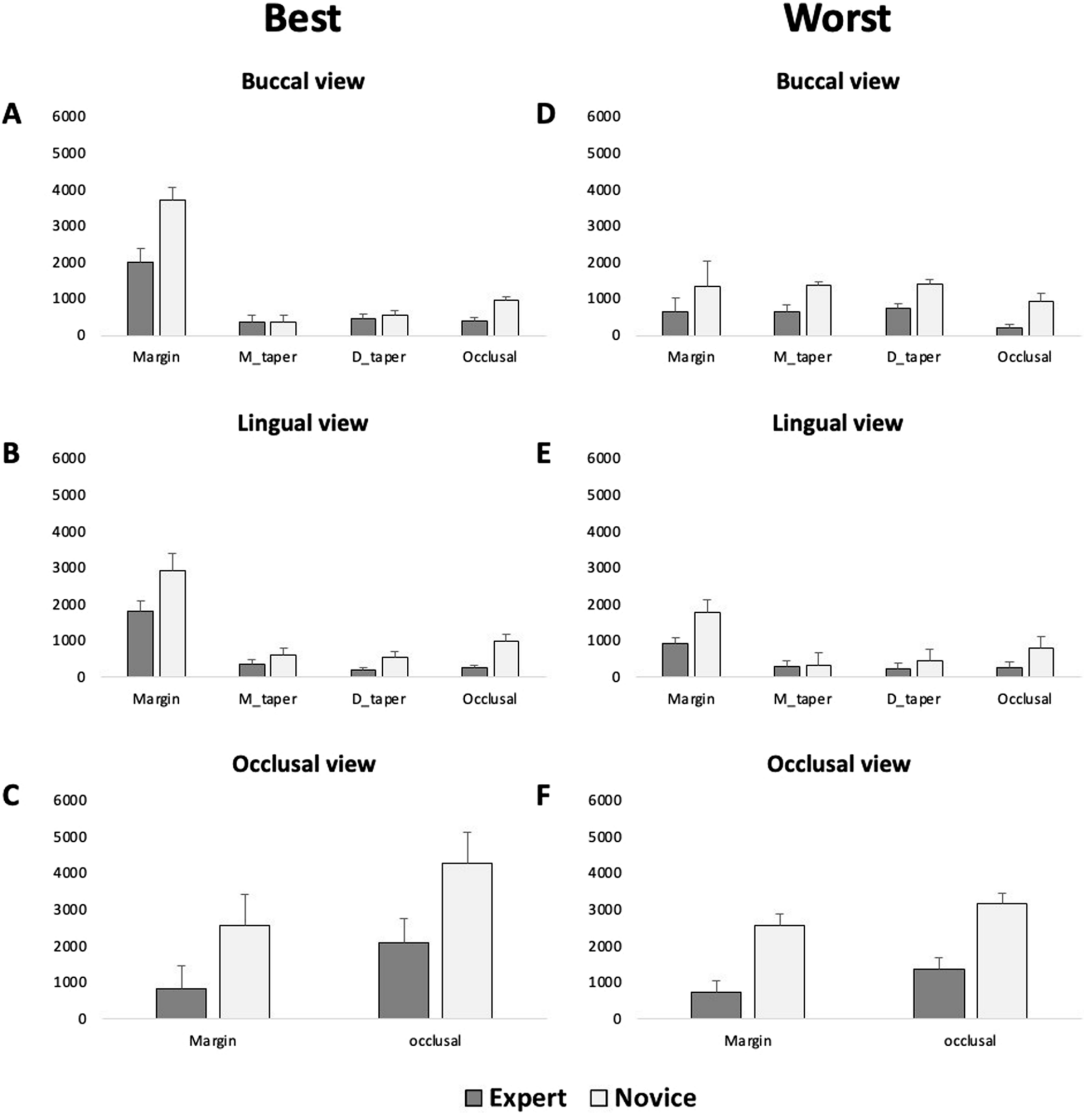
Mean and standard error mean of the total duration of fixation for the novice (dental students) and expert (dental educators) groups for buccal, lingual, and occlusal views for the best (**A**, **B**, **C**) and the worst (**D**, **E**, **F**) tooth preparation

The results of ANOVA analysis showed significant main effects of group (novices/experts) (*P* = 0.013) and condition (AOI: margin, mesial taper, distal taper, occlusal) (*P* < 0.050) but no significant effect of photos (best tooth preparation / worst tooth preparation) (*P* = 0.330). Post hoc analysis of the main effects of groups showed a significantly longer total duration of fixations in the novices compared to the experts. Post hoc analysis of the main effects of the condition showed a significantly longer total duration of fixations in the margin compared to all other conditions (*P* < 0.012). (Fig. 4).

The results of the ANOVA also showed significant interactions between condition and group (*P* = 0.015) and photos and condition (*P* < 0.001). Post hoc analysis of the condition and group showed a significantly higher total duration of fixations in the novices compared to the experts while observing the occlusion (*P* < 0.006). Post hoc analysis of the interaction between condition and photos showed significantly higher total duration of fixations in the best tooth preparation compared to the worst tooth preparation, while observing the margin (*P* < 0.001) and occlusion (*P* < 0.002). But post hoc analysis also showed a significantly higher total duration of fixations in the worst tooth preparation compared to the best tooth preparation, while observing the mesial taper (*P* < 0.002) but not the distal taper (*P* = 0.253).

### Lingual view

The result of ANOVA analysis showed significant main effects of group (novices/experts) (*P* = 0.006), photos (best tooth preparation / worst tooth preparation) (*P* = 0.020), and condition (AOI: margin, mesial taper, distal taper, occlusal) (*P* < 0.001). Post hoc analysis of the main effects of groups showed a significantly longer total duration of fixations in the novices compared to the experts (*P* < 0.007). Post hoc analysis of the main effects of photos showed a significantly longer total duration of fixation in the best tooth preparation compared to the worst tooth preparation (*P* < 0.03). Post hoc analysis of the main effects of the condition showed a significantly longer total duration of fixations in the margin compared to all other conditions (*P* < 0.001). (Fig. 4).

### Occlusal view

The result of the ANOVA analysis showed significant main effects of groups (novices/experts) (*P* < 0.001), photo (best tooth preparation / worst tooth preparation) (*P* = 0.008), and condition (AOI: margin, mesial taper, distal taper, occlusal) (*P* < 0.001). Post hoc analysis of the main effects of groups showed a significantly longer total duration of fixations in the novices compared to the experts (*P* < 0.001). Post hoc analysis of the main effects of the condition showed a significantly longer total duration of fixations in the occlusal area compared to the margin (*P* < 0.001). (Fig. 4) Post hoc analysis of the main effects of photos showed a significantly longer total duration of fixations in the best tooth preparation compared to the worst tooth preparation (*P* < 0.01).

The results of the ANOVA also showed significant interactions between groups and conditions (*P* = 0.048). Post hoc analysis of the condition and groups showed a significantly higher total duration of fixation in the novices compared to the experts while observing the margin (*P* < 0.001).

### Duration of assessment

The total time allocated to gaze at each of the pictures was 15 s and once the participants had observed the three views (buccal, lingual, and occlusal) they were asked to make the decision (if the preparation was ready for impression) and rate the tooth preparation (VAS).

The results of ANOVA analysis showed significant main effects of group (novices/experts) (*P* = 0.003), photos (best tooth preparation / worst tooth preparation) (*P* = 0.002), and views (buccal, lingual, and occlusal) (*P* = 0.012). Post hoc analysis of the main effects of groups showed a significantly longer time to decision in the novices compared to the experts. Post hoc analysis of the main effects of photos showed a significantly longer time to decision for both the groups in the best tooth preparation compared to the worst tooth preparation. Post hoc analysis of the main effects of the condition showed a significantly shorter duration of observation of the occlusal view compared to the buccal view.

**Fig. 5:**
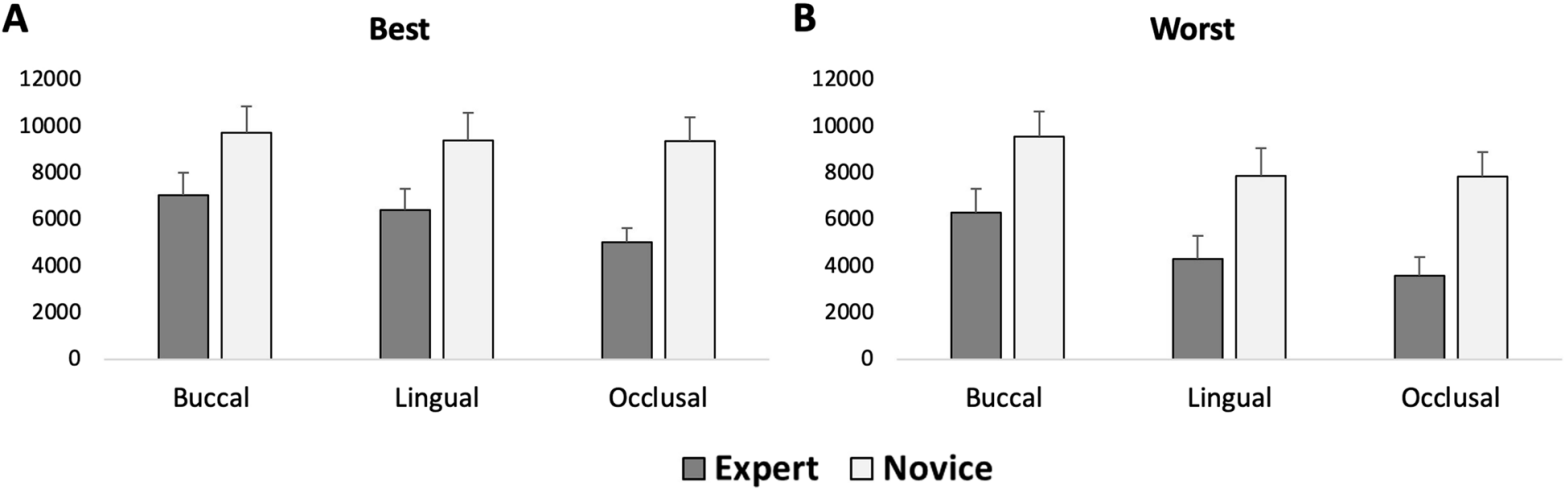
Mean and standard error mean of the duration of assessment for the buccal, lingual, and occlusal views for the best (**A**) and the worst (**B**) tooth preparation by the novice (dental students) and expert (dental educators) groups

The results of the ANOVA also showed significant interactions between photos and condition (*P* = 0.005). Post hoc analysis of the interaction between condition and photos showed a significantly higher total duration of observation in the best tooth preparation compared to the worst tooth preparation, while observing the lingual view (*P* < 0.001) and the occlusal view (*P* = 0.003) (Fig. 5).

## Discussion

Gaze behavior, measured through eye tracking, has been widely accepted as a key indicator of how humans process information from their surroundings and interact with the world [20]. As a result, and as mentioned above, eye tracking has emerged as a key tool in both clinical research and user experience research, as it can provide objective, quantitative data on visual attention, cognitive processes, and neurological function. In the current study, eye tracking was used to evaluate the differences in gaze behavior between novices comprising a group of undergraduate dental students and experts comprising qualified dentists while assessing a single crown tooth preparation. Specifically, both groups assessed precise AOIs on a buccal, lingual, and occlusal view of a “best” and “worst” tooth preparation. In accordance with the hypothesis, the results of the study showed significant differences in gaze behavior between undergraduate dental students (novice group) and dental educators (expert group) while evaluating a single crown tooth preparation. More specifically, the results of the study showed a significantly longer duration of fixation in all the three (buccal, lingual, and occlusal) views in the novices compared to the expert group. Overall, there were specific differences in the total duration of fixation between the novices and the experts, between best and worst preparations and also AOIs. The important interactions are discussed below.

Tooth preparation is the foundation of undergraduate dental education programs in dental fixed prosthetic restorations and the results of the current study may have an important implication for the undergraduate pedagogical training in dental education [21]. We believe that the current study is the first study that objectively analyzes the gaze behavior of participants while assessing tooth preparations. Overall, our goal is to provide educators with objective tools to better provide customized feedback to the undergrads in order to improve their abilities to self-assess their work in fixed prosthodontic education.

The design of the current study was such that the participants were asked to observe twenty different tooth preparations each with a buccal, lingual, and occlusal view on a computer screen. The participants were subsequently requested to evaluate the images and determine if the preparation was adequate for dental impression-making. The images were screenshots of scanned tooth preparations. The quality of the pictures displayed enabled participants to perceive the details of each preparation so that their assessment was as objective as possible in accordance to previous studies [22, 23]. Based on initial pilot testing it was decided that displaying 60 slides, each representing different views (buccal, lingual, and occlusal) of 20 different tooth preparations, provides a comprehensive and detailed assessment of the monolithic zirconia crown preparations. It was also observed that displaying 60 slides provided a balanced and comprehensive evaluation with practical considerations of time and efficiency, ensuring that all significant features of the tooth preparations were adequately covered while maintaining a manageable number of images for assessment. Also, each view captured unique details essential for accurate analysis, such as the contour, margin integrity, and overall quality of the preparation from different angles. It is also suggested that as participants viewed more images, they became increasingly familiar with the image quality, potentially enhancing their ability to assess the overall quality of various tooth preparations. In other words, because the participants viewed several images, they were more finely attuned to evaluating the overall quality of different tooth preparations. Recently, in a study involving spatial images, it was shown that imposing a time limit on participants highlighted differences in attention between novices and experts, whereas there was no difference without a time limit [24]. Accordingly, in the current study, the participants were given 15 s to observe each image and another 15 s to make the decision. In addition, the participants could also move on to the next image as soon as they had made their decision. Therefore, the study design is more robust to elucidate differences between the novice (i.e., undergraduate dental students) and experts (dental educators).

Studies have suggested that the total duration of fixation, a commonly used eye-tracking metric, is a useful tool in the study of learning processes [25]. Fixations are the moments when the eyes remain relatively still, focusing on a specific point, and are suggested to be essential for processing visual information [26]. In particular, the total duration of fixation measures the cumulative time spent fixating on specific areas of interest. It has been shown that the fixation durations of slower readers were typically shorter than those of skilled readers [27, 28]. Therefore, in the current study, it was decided to evaluate the total duration of fixation as a metric to evaluate the differences between the level of skills between the novices and experts.

The results of subject-based reports showed no significant difference in the visual analog scale ratings (and their dichotomous decision) between the novice and expert groups for either the best or worst tooth preparations. This finding implies that both groups had similar perceptions of tooth preparations, regardless of their professional background (undergraduate dental students vs. dental educators). However, they may have different perceptions of overall scores and the decision if they judged that the prepared tooth was ready to make an impression. There was also no correlation between the VAS scores and the dichotomous decision. This observation could be because the dichotomous decisions were perhaps not based on predefined criteria or thresholds, while the continuous scores perhaps represent a spectrum of values [29].

The results of the current study also showed that in general the time to decision was significantly longer while assessing the best picture compared to the worst picture for both novices and experts. However, the time to decision was significantly longer in novices compared to experts suggesting that undergraduate dental students took more time than dental educators to assess and decide on tooth preparations. These observations are in accordance with previous studies which suggested that novices needed more time and had a larger cognitive workload due to both the uncertainty and lack of experience compared to experts [11, 30–32].

The duration of assessment of the buccal view was longer than the occlusal view but not the lingual view. The buccal view seemed to allow participants to make a quick decision while assessing an unacceptable preparation, suggesting that they spent less time on the other views. It also appears that the buccal view is important while evaluating tooth preparation and that typically people take more time to evaluate a good preparation than a bad preparation. It is suggested that fixation is a metric to evaluate cognitive processing and longer fixations are generally interpreted as more processing [32]. Accordingly, we have observed that the participants tend to evaluate “obvious” discrepancies faster than the “not so obvious” discrepancies. Previous studies have shown that a greater number of fixations are indicative of greater visual attention. It has been shown that in general people fix their vision at a point of discrepancy without making any progress. Further, AOIs have been used in several studies involving a variety of medical specialties to define specific locations for eye-tracking software to provide gaze behavior information (duration of fixation, saccade, number of visits, etc.). [11, 14, 18, 33, 34]. In the current study, AOIs have been drawn according to the typical areas involved in the tooth preparation and its assessment (margin, mesial taper, distal taper, and occlusal) [35]. It was also observed that in general both the groups took considerably longer time to assess the margin than the other AOIs. This observation can be because the accurate placement and fit of the dental crown is dependent on the convergence of the mesial and distal walls and the overall shape of the preparation. Moreover, the mesial and distal taper AOIs could also be evaluated during the observation of the lingual view but the AOI “margin” for the buccal view can only be visible in the buccal view. If there are no obvious discrepancies in the mesial and distal walls people tend to evaluate the finishing line which is perhaps perceived as an important determinant of good tooth preparation and perhaps may be a difficult proposition to evaluate at a glance. Conversely, when an obvious undercut is present in the mesial and distal walls, participants tend to spot it quickly in the worst tooth preparation, regardless of the group they belong to, enabling faster decision-making. In contrast, in the best tooth preparation, participants tend to hesitate until they are certain of the absence of discrepancies, resulting in relatively longer decision-making times. Studies have suggested that a longer total duration of fixation indicates more visual attention and cognitive processing of the stimulus. Thus, the participants tend to spend more time fixating on areas that are more visually salient, informative, or cognitively demanding [36]. Thus, in the current study, the duration of fixation was longer for evaluating the margin and also for evaluating the best tooth preparation. These observations are in accordance with the previous observations that showed individuals tend to stare at a problem more without making any progress in relation to the task. It was also suggested that students often tend to spend a significant amount of time staring at a particular point which is perhaps an indication that they are uncertain about the next step. Therefore, a comprehensive evaluation of the total duration of fixation in eye-tracking metrics provides valuable insights into attention, cognitive processing, visual perception, perceptual load, and task demands. However, it's important to interpret this metric in conjunction with other eye-tracking measures and consider the specific context of the study or task to derive meaningful conclusions. Further, studies should confirm these statements by evaluating the cognitive implications on tooth-preparation assessment.

Acknowledging limitations in research studies is essential for understanding the boundaries and constraints inherent in the study design, data collection, and analysis. One such limitation in the current study is the use of corrective glasses. Previous research has suggested that corrective glasses can influence eye-tracking metrics. However, in the current study, we were unable to account for this variable. Authors from the previous studies have also highlighted limitations primarily concerning technical aspects, such as the eye-tracking device, lighting conditions, and the overall experimental design. However, the present study adhered to RESIDE recommendations, aiming to standardize parameters and minimize biases, which can be considered a strength of the study [19]. The results of the current study indicated that participants had longer fixation durations on the buccal view. It is suggested that the sequential display order of views (buccal, lingual, or occlusal) in the current study may introduce bias, as the buccal view consistently appeared first. Future studies could employ a randomized view in order to mitigate this potential bias. However, in the current study, the participants were exposed to a series of photographs under different conditions, followed by a selection of the “best” and “worst” rated photographs for the analysis which perhaps reduce biases.

## Conclusion

In summary, the results of the current study showed distinct differences in gaze behavior between the novice and the experts during the evaluation of single crown tooth preparation. In particular, the novice group of dental students showed a longer total duration of fixation across all the views (buccal, lingual, and occlusal) compared to the expert group of dental educators. Further, both novice dental students and expert dentists spent more time assessing the best tooth preparation compared to the worst tooth preparation. Yet, the novices showed a longer total duration of fixation than the experts in the assessment of both best and worst tooth preparations. Also, the margin seems to be the most important AOI in the assessment of single crown tooth preparation. The findings of the study may have implications for dental education and clinical practice. Understanding differences in gaze behavior between undergraduate dental students and dental educators could enhance diagnostic skills help improve tooth preparation skills and provide constructive feedback. Further analysis of fixation patterns and their association with clinical decision-making needs to be investigated.

## Data Availability

The datasets used and/or analyzed during the current study are available from the corresponding author upon reasonable request.

## Abbreviations

ANOVA: Analysis of variance
AOI: Area of interest
RESIDE: Reporting Eye-tracking Studies In Dentistry
VAS: Visual analog scale
